# Managing Psoriatic Arthritis With Inflammatory Bowel Disease and/or Uveitis

**DOI:** 10.3389/fmed.2021.737256

**Published:** 2021-09-16

**Authors:** Alfred Yu Ting Chia, Gladys Wei Xin Ang, Anita Sook Yee Chan, Webber Chan, Timothy Kit Yeong Chong, Ying Ying Leung

**Affiliations:** ^1^Duke-NUS Medical School, Singapore, Singapore; ^2^Translational Immunology Institute, SingHealth Duke-NUS Academic Medical Centre, Duke-NUS Medical School, Singapore, Singapore; ^3^Singapore National Eye Center and Singapore Eye Research Center, Singapore, Singapore; ^4^Department of Gastroenterology and Hepatology, Singapore General Hospital, Singapore, Singapore; ^5^Department of Rheumatology and Immunology, Singapore General Hospital, Singapore, Singapore

**Keywords:** psoriatic arthritis, uveitis, inflammatory bowel disease, co-morbidity, biologic therapy

## Abstract

Psoriatic arthritis (PsA) is a chronic inflammatory disease that presents with psoriasis (PsO), peripheral and axial arthropathy. The heterogeneity of disease presentation leads to the term “psoriatic disease (PsD)” which is thought to better encompass the range of clinical manifestations. PsA is associated with several comorbidities such as cardiovascular diseases, metabolic syndrome and other extra-articular manifestations including uveitis, and inflammatory bowel disease (IBD). While novel therapeutics are being developed following advances in our understanding of the pathogenesis of the disease, the diverse combinations of PsA with its various comorbidities still pose a clinical challenge in managing patients with PsA. This article reviews our current understanding of the pathogenesis of PsA and how various pathways in the pathogenesis lead to the two comorbid extra-articular manifestations – uveitis and IBD. We also review current evidence of treatment strategies in managing patients with PsA with comorbidities of uveitis and/or IBD.

## Introduction

Psoriatic arthritis (PsA) is an inflammatory arthritis associated with psoriasis (PsO) (1). It belongs to the family of spondyloarthritis (SpA) and the musculoskeletal manifestations include peripheral arthritis, dactylitis, enthesitis, and axial arthropathy. The impact of PsA extends beyond skin and joints to disability, fatigue, anxiety, depression, and poor quality of life (2, 3). PsA is associated with comorbidities such as obesity, metabolic syndrome, insulin resistance, and cardiovascular disease (4). The extra-articular manifestations of PsA include inflammatory bowel disease (IBD) and uveitis (5). In recent years, there are advancements in therapeutic options to treat musculoskeletal manifestations of PsA (6), but research to understand the pathogenesis of extra-articular manifestations and their treatment options is still in infancy. The purpose of this review is to summarize the current understanding of pathogenesis of PsA and the extra-articular manifestations and their treatment options.

## Extra-Articular Manifestations

### IBD

Crohn's disease (CD) and ulcerative colitis (UC) are the two main forms of IBD. CD is characterized by chronic, patchy granulomatous inflammation with skip lesions, affecting any part of the gastrointestinal tract, especially the terminal ileum and colon. The inflammation is transmural which can lead to fibrosis, stricture, and fistula. In contrast, UC is characterized by continuous mucosal inflammation extending from the rectum proximally toward the colon. Differentiating these two conditions is important as each has diverse prognoses and differential responses to treatment (7). The clinical presentations of IBD include recurrent abdominal pain, bloody diarrhea, and mucus in the stool. Patients with CD can present with intestinal obstruction, recurrent fistulas, and other perianal findings. Systemic symptoms include fatigue, weight loss, fever, and symptoms of anemia. The standardized mortality ratio for CD ranges from 1.2 to 1.9 times the general population (8). The prognosis of IBD has improved in recent decades due to therapeutic advances.

Amongst patients with IBD, extraintestinal manifestations are common, including musculoskeletal (axial and peripheral arthropathy and arthritis), ocular (uveitis, scleritis and episcleritis), and skin. Inflammatory arthropathies are reported up to 40% of patients with IBD (9). While asymptomatic sacroiliitis may be seen in up to three-quarters of IBD patients, the reported prevalence of seronegative SpA ranges from 18–45%, and ankylosing spondylitis (AS) 3–9.9% (10, 11). Peripheral arthritis is reported in 7–16% of IBD patients. Peripheral arthritis is mainly asymmetrical and oligoarticular, usually acute and occurs during IBD exacerbations, and self-limiting. However, it may also persist for months or years. Its onset usually coincides with or after IBD but may precede IBD. Enthesitis and dactylitis were reported in 2–4% of patients (12).

Amongst patients with SpA, IBD is common (13). Patients with PsO, PsA and AS have a 1–4 fold increased risk of IBD compared to the general population (14–18). Among patients with SpA, 30–42 % have endoscopic (macroscopic) gastrointestinal inflammation (19–22) while 46–58 % have histologic (microscopic) inflammation (20, 21, 23). The presence of macroscopic or microscopic inflammatory lesions poorly correlates with symptoms (19). In patients with axial SpA, the severity of microscopic inflammation was significantly associated with severity of bone marrow edema on magnetic resonance imaging, indicating a link between mucosal inflammation and progressive disease (24).These subclinical gastrointestinal inflammatory lesions may predispose SpA patients to develop IBD, with a lifetime IBD risk of between 4–8% (25–28). Among patients with PsO and PsA, IBD is more common in patients with more severe PsA (29). IBD is also more common in patients with axial-PsA than in those with peripheral-only PsA (30).

### Uveitis

Uveitis is the inflammation of the uveal tract of the eye which comprises of the iris, ciliary body, and choroid. Adjacent structures including retina, optic nerve, vitreous, and sclera may also be affected. Clinically, uveitis is categorized anatomically – anterior, intermediate, posterior, or panuveitis (31). There is an increased association of ocular manifestations amongst patients with PsD (32, 33). Other presentations like vitritis, retinal vasculitis, and cystoid macular edema involving the posterior chamber are sight-threatening (34, 35). The prevalence of uveitis increases with disease duration, lifelong prevalence is over 40%. Among patients with SpA, acute anterior uveitis (AAU) is most common (26) and its prevalence varied with the type of SpA: 33% in AS; 37% in IBD-associated arthritis; 26% in reactive arthritis; 25% in PsA; and 13% in undifferentiated SpA (36, 37). In both Asian and Western populations, uveitis is common in patients with severe PsO and those with PsA (38, 39). Uveitis in SpA usually presents with a ‘unilateral alternating' pattern, sudden in onset, confined to the anterior chamber, and completely resolves between episodes (40). In contrast, uveitis in PsA is insidious in onset, bilateral with a chronic relapsing course. PsA patients with both uveitis and axial arthropathy tend to be male and HLA-B^*^27 positive (41, 42). HLA-DR^*^13 positivity is also associated with uveitis in patients with PsA (43). Uveitis may also precede the development of PsA in patients with PsO (44).

## Pathogenesis

### Pathogenesis of PsA

**Figure 1:**
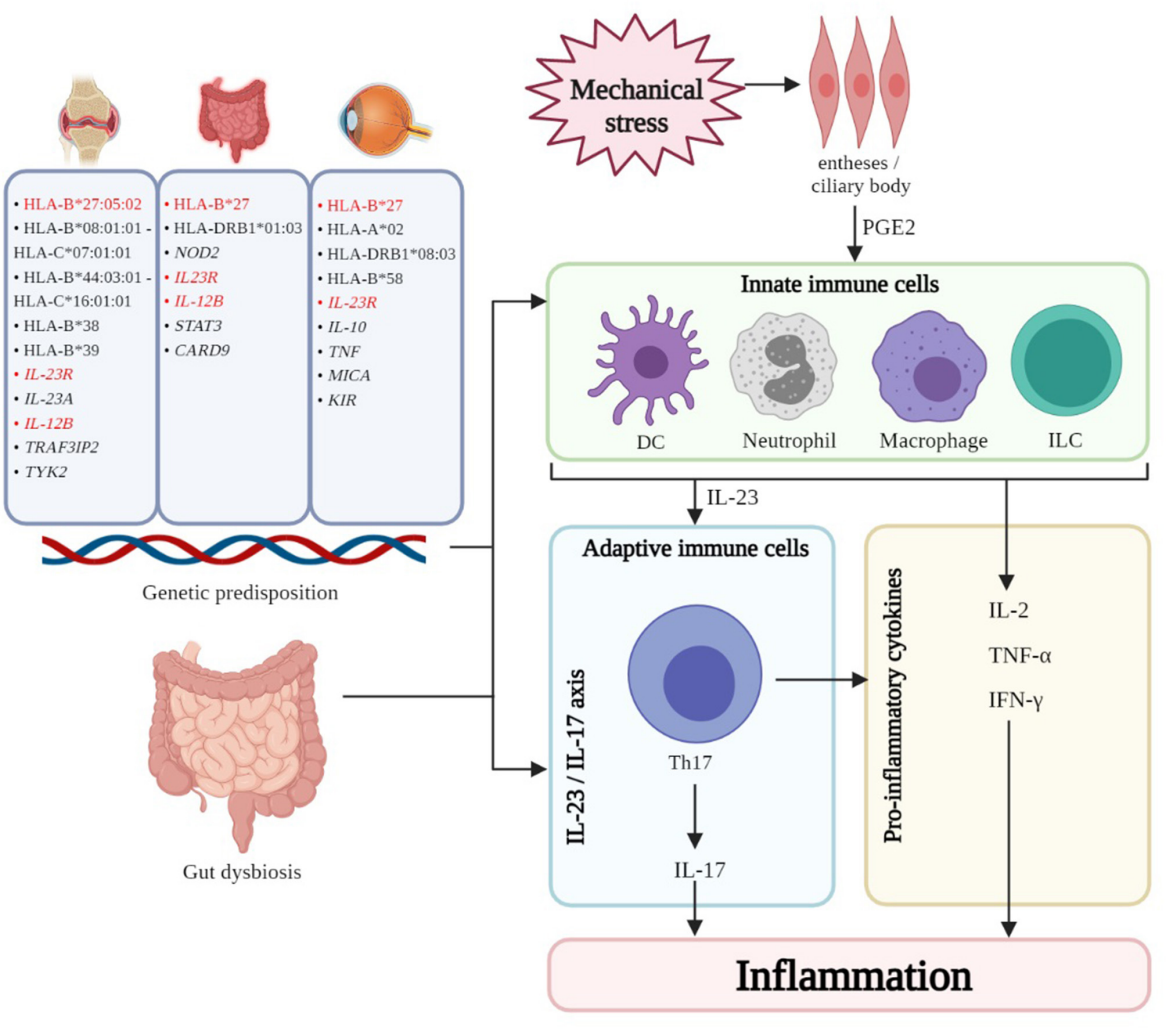
The interplay of genetic, immune, and other factors results in inflammation of the various domains - skin, joints, gut, and eye - in PsD. Common genetic associations (highlighted in red) can be found amongst the three manifestations. Gut dysbiosis is thought to contribute to the pathogenesis for the three manifestations by leakage of bacterial antigen into systemic circulation thereby resulting in inflammation and/or trafficking of immune subsets to and from the GI mucosa and other sites. In PsA enthesitis and uveitis, mechanical stress triggers the release of PGE2, resulting in the recruitment and activation of innate immune cells (DC, neutrophils, macrophages, ILC like type 3 ILC, MAIT cells, γδ T cells) which perpetuate inflammation. Furthermore, these innate cells secrete cytokines, notably IL-23, which polarize and maintain Th17 cells which are central to the IL-23/IL-17 axis which is believed to be important in the pathogenesis of PsD. HLA, human leukocyte antigen; *IL-23R*, interleukin-23 receptor; *IL-23A*, interleukin-23A; *IL-12B*, interleukin-12B; *TRAF3IP2*, tumor necrosis factor receptor-associated factor 3-interacting protein 2; *TYK2*, tyrosine kinase 2; *NOD2*, nucleotide-binding oligomerization domain-containing protein 2; *STAT3*, signal transducer and activator of transcription 3; *CARD9*, caspase recruitment domain family Member 9; *TNF*, tumor necrosis factor; *MICA*, major histocompatibility complex class I chain-related gene A; *KIR*, killer immunoglobulin receptor; DC, dendritic cell; ILC, innate lymphoid cell; MAIT, mucosal-associated invariant T cell; Th17, T helper 17 cell; IL-17, interleukin-17; IL-2, interleukin-2; TNF-α, tumor necrosis factor alpha; IFN-γ, interferon gamma. Created with BioRender.com.

A combination of genetic and environmental factors contributes to pathogenesis of PsA (Figure 1). Genetic component in PsA is strong (45). HLA class I alleles such as HLA-B^*^27:05:02 haplotype is widely reported to be positively associated with enthesitis, dactylitis, and sacroiliitis while the HLA-B^*^08:01:01–HLA-C^*^07:01:01 haplotype is positively associated with joint fusion, deformity and asymmetrical sacroiliitis. In contrast, the B^*^44:03:01–C^*^16:01:01 haplotype may be protective against enthesitis (46). Additional HLA haplotypes associated with susceptibility to PsA were HLA-B^*^38, and HLA-B^*^39 (47–51). Non-HLA PsA susceptibility loci related to inflammatory pathways have been implicated. IL-23 receptor (*IL-23R*) polymorphisms are associated with risk of PsA (52). Tumor necrosis factor receptor-associated factor 3-interacting protein 2 (*TRAF3IP2*), encoding nuclear factor-κB (NFκB) activator protein 1 (Act1) which is an adaptor protein for interleukin-17 (*IL-17*) receptor (53–55), *IL-23A, IL-12B*, and *TYK2* (Tyrosine Kinase 2) are other examples, highlighting the importance of IL-23/IL-17 axis in the pathogenesis of PsA (56).

In a genetically predisposed individual, environmental factors including mechanical stress may trigger enthesitis – a hallmark clinical presentation of SpA including PsA (57, 58). Mechanical stress and trauma release damage-associated molecular patterns (DAMPs), triggering the production of prostaglandin E2 (PGE2) (59) by resident mesenchymal cells, which recruit innate immune cells to perpetuate inflammation. PGE2 also induces T cell secretion of IL-17, a key driver in PsA pathogenesis (58, 60). Innate immune cells such as dendritic cells (DCs), monocytes/macrophages, neutrophils, and innate lymphoid cells (ILCs) corroborate with adaptive immune cells to perpetuate inflammation in PsA (61). Additionally, plasmacytoid dendritic cells (pDCs) infiltrate the synovium to act as antigen presenting cells (APCs), triggering downstream expression of TNF-α, IFN-γ, and IL-2 from CD68+ macrophage-like-synoviocytes that mediate synovial inflammation and bone erosions (62, 63). TNFα synergizes with DCs to activate and polarize Th17 cells (64). In addition to Th17 cells, type 3 innate lymphoid cells (ILCs) (65, 66), mucosal-associated variant T (MAIT) cells (67, 68), and γδ T cells (69) are recruited to the synovium and produce IL-17A upon stimulation (70). In short, the IL-23/IL-17 axis is the central driver of inflammation in PsA (71–75).

### Pathogenesis of IBD

Genetic predisposition increases the risk of developing IBD amongst patients with PsA and SpA. HLA-B27 is the major HLA associated with IBD risk. 25–78% of patients with AS and IBD are HLA-B27 positive (9, 76, 77). HLA-DRB1^*^01:03 is also common between AS and IBD (76, 78–80). Non-HLA polymorphisms such as nucleotide-binding oligomerization domain-containing protein 2 (*NOD2)* polymorphisms increase the risk of CD about 4–40 times and is associated with sacroiliitis amongst patients with IBD. NOD2, an intracellular receptor expressed by immune and intestinal cells, is involved in the activation of NFκB and inducing pro-inflammatory genes in innate immune cells (81–85). *IL-23R* polymorphisms modify susceptibility to IBD, where a loss-of-function mutation may have protective effect against IBD (86). Polymorphisms in genes related to the IL-23/IL-17 axis such as *IL-12B*, signal transducer and activator of transcription 3 (*STAT3*), and caspase recruitment domain family member 9 (*CARD9*) are associated with CD (87). Once again, this highlights the IL-23/IL-17 axis as a major pathogenetic pathway for IBD manifestations in patients with PsA.

The microbiome plays an important role in gastrointestinal health, and dysbiosis of the microbiota is observed in patients with IBD. Microbiota in IBD patients is less diverse compared to healthy controls. Gastrointestinal bacteria may invade the sterile inner colonic adherent mucus layer, disrupt epithelial architecture, and allow leakage of bacterial antigen into the systemic circulation to induce and perpetuate inflammation (88–90). A “gut-joint axis” has been proposed where immune cells traffic between the two domains (91, 92). Fecal microbiota transplantation (FMT) has shown promising results in the treatment of UC in a Cochrane Database systematic review (93). Positive clinical outcomes are associated with higher dosage and delivery of FMT via lower gastrointestinal tract (94), and may be dependent on stool donor (95). However, a recent RCT on FMT in 31 patients with active PsA randomized to FMT vs. sham treatment was not efficacious for arthritis (96). Further study is required.

In patients with IBD, the number of IL-17-secreting MAIT cells (97), was increased in the gastrointestinal tract as compared to the peripheral blood, echoing PsA studies showing depleted MAIT cells in blood, and increased MAIT cells in inflamed synovia and psoriatic skin (67, 68). γδ T cells are found in colonic mucosa and represent around 40% of intraepithelial lymphocytes (98). In contrast to PsA, the presence of γδ T cells appears to be protective and anti-inflammatory in patients with IBD. Different subtypes of γδ T cells may behave differently in different cytokine environments, explaining the diverse observations of γδ T cells in PsA and IBD (99, 100). As with PsA, Th17 cells are major players in IBD (101). The chemokine receptor CCR6 is the main surface marker of the Th17 lineage. CCL20, a ligand for CCR6, is elevated in IBD gut epithelium and likely contributes to recruitment of CCR6+ type 3 ILC, Th17, and dendritic cells (102, 103). Due to high levels of IL-17 and IL-23 in IBD gut epithelia, the IL-23/IL-17 pathway was thought to be a therapeutic target (104–106). However IL-23 inhibition showed efficacy in patients with IBD but IL-17 inhibition lead to disease exacerbation (107). A possible explanation for this paradox is that IL-17 plays a role in maintaining intestinal barrier and microbial defense (108–110).

### Pathogenesis of Uveitis

The HLA-B^*^27 is associated with increased risk of AAU (111), and is a common risk locus for PsA (and other SpA) and uveitis (112). HLA haplotypes such as HLA-A^*^02 (113), HLA-DRB1^*^08-03 (114), HLA-B^*^58 (115) were also associated with development of uveitis. Other non-HLA susceptibility loci are major histocompatibility complex class I chain-related gene A (*MICA*) (116, 117), IL-10 (118), *TNF* (119), killer immunoglobulin receptor (*KIR*) (120), and polymorphisms in *IL-23R*, which all participate in immune response (121). Nonetheless, positive risk polymorphisms do not necessarily translate to uveitis. Other environmental and undiscovered factors are likely required to initiate uveitis in patients with SpA.

The eye is an immunologically privileged organ with a local inhibitory microenvironment, entailing immune ignorance and tolerance to prevent inflammation. The blood-retina barrier and absence of efferent lymphatics reduces exposure of the eye to the circulating immune system (122). In uveitis, infiltration of immune cells into the eye and disrupts the immunologically quiescent environment. However, the trigger of this infiltration is undetermined (123). Some evidence implicates the perturbation of the gut microbiome to SpA-associated uveitis. Animal studies demonstrated trafficking of leucocytes from intestine to eye, supporting the concept of a gut-eye axis (124). Further evidence from retina-specific TCR transgenic mice reared under germ-free conditions showed that the severity of uveitis was reduced in the absence of gut microbiota. This reduction of severity was associated with a reduction in Th17 cells in the lamina propria of the intestine. Reconstitution of gut microbiota increased retina-specific T cell signaling (125). McGonagle *et. al* (2015) proposed that anterior uveal structures are analogous to entheses due to their mechanical and structural roles in lens suspension. The repeated contractions and relaxations of these structures expose them to mechanical stress just like musculoskeletal entheses, thus providing the initial stimuli for inflammation (126). Like entheseal mesenchymal cells in enthesitis, cells in ciliary body express IL-23R, suggesting receptiveness to IL-23 signaling (127). In patients with uveitis, serum IL-17A levels were elevated during active disease (128). Association between Th17 and the development of uveitis has been observed in animal and clinical studies highlighting the importance of the IL-23/IL-17 axis in driving inflammation in PsA and uveitis (129–132). However, clinical trials have yet to demonstrate the efficacy of IL-17 inhibition in uveitis (133).

## Management of Extra-Articular Manifestations in PSA

Therapeutic advances in the last decade for PsA and PsO have improved quality of life of many. The European League Against Rheumatism (EULAR) developed algorithm treatment recommendations for the musculoskeletal manifestations of PsA (134). However, patients who have co-existing non-musculoskeletal manifestations such as IBD and uveitis pose a clinical challenge. The Group for Research and Assessment of Psoriasis and Psoriatic Arthritis (GRAPPA) recommendation guideline highlighted a domain-driven approach focused on peripheral, axial, dactylitis, enthesitis, skin and nails (135, 136). The evidence for optimal treatment options for extra-articular manifestations in PsA is lacking and relies on evidence built in the fields of IBD and uveitis as independent conditions. Regardless of domains, the treatment goals are moving toward achieving low or minimal disease activity. Although some treatment options are common across the different domains, the doses may be different. We summarize the usual doses used for various domains in Table 1.

**Table 1:** Therapeutic options and common dosing regimen for PsD and extra-articular manifestations.

**Drug class**	**Agents**	**Dosage for domains**				
		**Peripheral arthritis**	**Axial arthritis**	**Psoriasis**	**IBD**	**Uveitis**
Corticosteroid		- Intra-articular corticosteroid injection as indicated - Systemic corticosteroid to be avoided	- No indication	- Topical corticosteroid - Systemic corticosteroid to be avoided	- Induction: corticosteroid short tapering course- Maintenance: not indicated	- Corticosteroid eye drops tapering course - Periocular corticosteroid injections or intravitreal implants - Systemic corticosteroid for sight-threatening disease
Immune-modulator	Methotrexate Sulfasalazine Leflunomide Cyclosporin Thiopurines	- MTX 10–25 mg QW, PO - SSZ 500 mg-3g/day, PO - LEF 10–20 mg OM, PO - CyA 2.5-4 mg/kg/day, PO	- Not effective	- MTX 10–25 mg QW, PO - CyA 2.5–5 mg/kg/day, PO	- 5-ASA (UC): 1.5–4.5 g/day, PO- MTX 25 mg QW, SC or IM- SSZ 3–4 g/day, PO- AZA 2.5 mg/kg, PO	- MTX 7.5–20 mg QW, PO - SSZ 3–4 g/day, PO
TNFI	Infliximab	- 5–10 mg/kg loading at W0, 2 and 6, IV - then Q6–8W, IV	- 5–10 mg/kg loading at W0, 2 and 6, IV- then Q6–8W, IV	- 5–10 mg/kg loading at W0, 2 and 6, IV - then Q8W, IV	- Induction (CD/UC): 5–10 mg/kg loading at W0, 2 and 6, IV- Maintenance (CD/UC): 5–10 mg/kg Q8W, IV	Off label use - Induction: 4 to 6 mg/kg at W0, 2, 6, then Q4W until clinical remission, IV - Maintenance: 5 mg/kg Q10–12W, IV
	Adalimumab	- 40 mg Q2W, SC	- 40 mg Q2W, SC	- 40 mg Q2W, SC	- 160 mg or 80 mg at W0, then 80 mg at W2, then 40 mg Q2W, SC	- 40 mg Q2W, SC
	Etanercept	- 50 mg QW to BIW, SC^*^	- 50 mg QW, SC	- 50 mg BIW for 3 months, - then 50 mg QW, SC^*^	- 25 mg BIW, SC^*^	- No indication
	Golimumab	- 50 mg Q4W, SC - Or 100 mg Q4W, SC if BW >100 kg - Alternative IV formulation at 2 mg/kg at W0 and W4, then Q8W	- 50 mg Q4W, SC	Off label use - Not primary approved for Psoriasis	- Induction (CD/UC): 200 mg at W0, then 100 mg at W2- Maintenance (CD/UC): 100 mg Q4W	- Off label use
	Certolizumab	- 400 mg at W0, 2 and 4, then 200 mg Q2W, SC - 400 mg Q4W, SC can be considered for maintenance	- 400 mg at W0, 2 and 4, then 200 mg Q2W, SC- 400 mg Q4W, SC can be considered for maintenance	- 400 mg Q2W, SC - For BW <90 kg, can consider 400 mg at W0, 2 and 4, then 200 mg Q2W, SC	- Induction (CD/UC): 400 mg at W0, 2, 4, SC- Maintenance (CD/UC): 400 mg Q4W, SC	- Ongoing phase III trial, promising preliminary data - 400 mg at W0, 2, 4; then 200 mg Q2W
IL-17i	Secukinumab	- Loading 150 mg QW for 5 doses, then monthly, SC - (300 mg if TNFi experienced)	- Loading 150 mg QW for 5 doses, then monthly, SC	- Loading 300 mg QW for 5 doses, then monthly, SC	- (CD) no difference compared to placebo, more adverse events, not indicated	Failure in 3 RCTs - Higher dose is superior to lower doses - No indication
	Ixekizumab	- Loading 160 mg once, then 80 mg monthly, SC	- Loading 160 mg once, then 80 mg monthly, SC	- 160 mg at W0, then 80 mg at W2, 4, 6, 8, 10, and 12, then 80 mg Q4W, SC	- No study, no indication	- No study, no indication
IL-12/23i	Ustekinumab	- 45 mg Q4W for 2 doses, then Q12W - 90 mg Q4W for 2 doses, then Q12W if BW>100 kg	- No indication	- 45 mg Q4W for 2 doses, then Q12W - 90 mg Q4W for 2 doses, then Q12W if BW>100 kg	- Induction: single weight-based dose (<55 kg, 260 mg, 55–85 kg, 390 mg, >85 kg 520 mg), IV- Maintenance: 90 mg Q8W, SC	- Ongoing phase 2 trials
IL-23i	Guselkumab	- Loading 100 mg Q4W for 2 doses, then 100 mg Q8W, SC	- No indication	- Loading 100 mg Q4W for 2 doses, then 100 mg Q8W, SC	- Ongoing phase II/III RCTs	- No study, no indication
	Risankizumab	- 150 mg Q4W for 2 doses, then Q12W, SC	- No difference compared to placebo, no indicated	- 150 mg Q4W for 2 doses, then Q12W, SC	- Ongoing phase III studies in CD- Induction (CD): 600 mg or 1200 mg once, IV- Maintenance (CD): 600 mg or 1200 mg Q12W, SC	- No study, no indication
α4β7 integrin inhibitor	Vedolizumab	- No indication	- No indication	- No indication	- Induction (CD/UC): 300 mg at W0, 2, and 6, IV- Maintenance (CD/UC): 300 mg Q8W, IV	- No indication
JAKi	Tofacitinib	- 5 mg BD, PO	- 5 mg BD, PO	- 10 mg BD, PO	- Induction (UC only): 10 mg BD for at least 8 weeks; PO- Maintenance (UC only): then 5 or 10 mg BD, PO- CD: No difference compared to placebo, - No indication	- No study, no indication
	Upadacitinib	- 15 mg OM, PO	- 15 mg OM, PO	- 15 mg OM, PO	- Phase II dose ranging RCT in CD	- No study, no indication

* *Less favored due to lower efficacy; not yet approved by authorities; 5-ASA, 5-aminosalicylic acid; AZA, azathioprine; BD, two times per day; BIW, twice per week; BW, body weight; CD, Crohn's disease; CyA, cyclosporin A; IL, interleukin; IM, intramuscular; IV, intravenous; OM, daily; PO, per oral; JAKi, Janus kinase inhibitors; LEF, leflunomide; MTX, methotrexate; Q, every; SC, subcutaneous; SSZ, sulfasalazine; TNFi, tumor necrosis factor inhibitors; UC, ulcerative colitis; W, week*.

### Therapeutic Goals

The treatment targets for patients with IBD are clinical remission, mucosal healing, and restoring quality of life (137, 138). The importance of mucosal healing defined as restitution of the intestinal lining and regression or disappearance of endoscopic lesions has been emphasized. Achievement of this target is associated with reduced risk of relapse, reduced hospitalization rates, steroid-free remission, and resection-free status (139–141). With medical advancements, the need for bowel resection is substantially reduced (142).

### Medical Therapies for CD

Corticosteroids can be used to induce clinical remission. It is given either topically as ileal-release budesonide for active mild-to-moderate CD or systemically for moderate-to-severe CD (132). However, systemic corticosteroid should not be used for maintenance (143, 144). Early initiation of corticosteroid-sparing immunomodulators such as azathioprine (AZA), mercaptopurine or methotrexate (MTX) for maintenance should be considered, although the level of evidence supporting efficacy of these drugs is relatively low (144, 145).

Monoclonal antibody targeting TNFα (TNF inhibitors, TNFi) has become the standard of care for patients with moderate-to-severe, active CD. Infliximab (IFX), adalimumab (ADA), and certolizumab (CZP) have demonstrated efficacy in inducing remission and maintenance in RCTs, and well supported by meta-analyses (146, 147). We summarized the major RCTs supporting the efficacies of TNFi in IBD Table 2. In a Cochrane Database Systematic review, CD patients who responded to induction by TNFi were more likely to maintain remission at 52 weeks with TNFi compared to placebo (147). Continued treatment with TNFi reduces surgery and hospitalization for CD (168, 169). Combination therapy of IFX with AZA was more efficacious than either agent alone in achieving response, inducing clinical and histological remission (156), suggesting synergistic effect. TNFi appears to be more effective when given at earlier stage of disease, with higher rates of response and remission, than given at later stage of disease (170, 171). Early escalation to TNFi treatment should be considered for patients with extensive disease and poor prognostic factors (144, 145).

**Table 2:** Evidence from major clinical trials for class of therapeutic options for Crohn's disease.

**Class of drug**	**Agent**	**Trial acronyms**	**RCT Phase**	**Sample size**	**Patient population**	**Treatment vs. comparison**	**Outcomes**
TNFi	ADA	CLASSIC I (148)	II	299	Active CD, naïve to TNFi (induction)	- ADA 160/80 mg - Vs. 80/40 mg, - Vs. 40/20 mg at W0, 2, SC	- Clinical remission at W4: ADA 160/80 36% (*p =* 0.001), ADA 80/40 24% (*p =* 0.06), ADA 40/20 18% (*p =* 0.036), Vs. PBO 12% (all comparison vs. PBO).
	ADA	CLASSIC II (149)	II	276	CD achieved induction in CLASSIC I (maintenance)	Patients achieved remission in CLASSIC I were re-randomized (*n =* 55) to - ADA 40 mg QW, SC - Vs. ADA 40 mg Q2W, SC - Vs. PBO - Patients not achieved remission (*n =* 209) received open-labeled ADA 40 mg Q2W, SC	- Clinical remission at W56 for re-randomized (*n =* 55): ADA 40 mg QW 83%, ADA 40 mg Q2W 79% Vs. PBO 44% (all *p <* 0.05 vs. PBO). - Clinical remission at W56 for open labeled patients (*n =* 209): ADA 46%
	ADA	GAIN (150)	III	325	Active CD, TNFi IR (induction)	ADA 160 mg at W0, then 80 mg at W2, SC vs. PBO	- Clinical remission at W4: ADA 21.6% vs. PBO 6.7%, (*p <* 0.001).
	ADA	CHARM (151)	III	854	Active CD despite immunomodulators, non-TNFi IR. All received open labeled induction: ADA 80 mg at W0, 40 mg at W2, SC (maintenance)	Maintenance: - ADA 40 mg Q2W, - vs. 40 mg QW - vs. PBO	- Clinical remission at W26: ADA QW 47% vs. ADA Q2W 40% vs. PBO 17% (all *p <* 0.001 vs. PBO). - Clinical remission at W56: ADA QW 41% vs. ADA Q2W 36% vs. PBO 12%, (all *p <* 0.001 vs. PBO).
	ADA	EXTEND (152)	III	129	Active CD, responded to open labeled ADA induction (160/80 mg at W0, 2, SC) at W4 (maintenance)	Maintenance: - ADA 40 mg QW, SC - vs. 40 mg Q2W, SC - vs. PBO	- Mucosal healing at W12: ADA 27% vs. PBO 13%, (*p =* 0.056). - Clinical remission at W12: ADA 52% vs. PBO 28% (*p =* 0.006) - Mucosal healing at W52: ADA 24% vs. PBO 0% (*p <* 0.001). - Clinical remission at W52: ADA 28% vs. PBO 3% (*p <* 0.001).
	IFX	(153)	II	108	Moderate to severe CD, naïve to TNFi (induction)	- IFX 5 mg/kg, once, IV - vs. IFX 10 mg/kg, once, IV - vs. IFX 20 mg/Kg, once, IV - vs. PBO	- Clinical response W4: IFX 5 mg 81% (*p =* 0.33) vs. 10 mg 50% (*P =* 0.26) vs. 20 mg: 64% (*p =* 0.01) vs. PBO 17%. (all comparison vs. PBO) - Clinical remission W4: IFX (all doses) 33% vs. PBO 4% (*p =* 0.005).- Clinical response W12: IFX (all doses) 41% vs. PBO 12% (*p =* 0.008).
	IFX	ACCENT-I (154)	III	573	Active CD, despite immunomodulators naïve to TNFi, all received open-labeled IFX induction, then re-randomized for maintenance (induction and maintenance)	Open-labeled induction (all): IFX 5 mg/kg at W0, IV par Randomized at W2 for maintenance: - IFX 10 mg/kg at W2, 6, then Q8W, IV - Vs. IFX 5 mg/kg at W2, 6, then 5 mg/kg Q8W, IV - Vs. PBO	- Induction: 58% responded to initial IFX at W2. - Clinical remission at W30: IFX10 mg/kg 45% (*p =* 0.003), vs. IFX5 mg/kg 39% (*p =* 0.0002), vs. PBO 21%. (all comparison vs. PBO) - Median time to loss of response at W54: IFX 10 mg/kg >54W (*p =* 0.002), vs. IFX 5 mg/kg 38W (*p =* 0.0002), vs. PBO 19W (all comparison vs. PBO)
	IFX	ACCENT-II (155)	III	282	Fistulating CD, naïve to TNFi (induction and maintenance)	Open-labeled induction (all): IFX 5 mg/kg at W0, 2, 6, IV - Randomized at W14 for maintenance: - IFX 5 mg/kg Q8W, IV - vs. PBO	- Induction: 69% responded to initial IFX at W14. - Time to loss of response: IFX >40W vs. PBO 14W (*p <* 0.001) - Clinical response W54: IFX 36% vs. PBO 19% (*p =* 0.009)
	IFX	SONIC (156)	III	508	Active CD, naïve to immunomodulator and TNFi (induction and maintenance)	IFX 5 mg/kg at W0, 2, 6, then Q8W + AZA 2.5 mg/kg/day - vs. IFX alone - vs. AZA alone	- Corticosteroid-free remission W26: IFX+AZA: 57% (*p =* 0.002 vs. IFX; *p <* 0.001 vs. PBO), vs. IFX alone: 44% (*p =* 0.006 vs. AZA), vs. AZA alone: 30%. - Mucosal healing W26: IFX+AZA 44% (*p =* 0.06 vs IFX; *p =* <0.001 vs. AZA), vs. IFX alone 30% (*p =* 0.02 vs. AZA), vs. AZA alone: 17%.
	CZP	PRECiSE I (157)	III	662	Active CD, 17% concomitant corticosteroid and immunomodulators, 28% prior TNFi (induction)	- CZP 400 mg at W0, 2, 4, then Q4W, SC - vs. PBO	- Clinical response at W6: CZP 35% vs. PBO 27%, (*p =* 0.02); - Clinical response at both W6 and W26: CZP 23% vs. PBO 16%, (*p =* 0.02) - Remission at W6: CZP 14% vs. PBO 10% (*p =* 0.17) - Remission at both W6 and W26: CZP 22% vs. PBO 17% (*p =* 0.07)
		PRECiSE II (158)	III	428	Active CD, 24% concomitant corticosteroid and immunomodulators, 15% prior TNFi (maintenance)	Open-labeled induction (*n =* 668): CZP 400 mg at W0, 2, 4, SC Patient with clinical response were randomized at W6 for maintenance (*n =* 428): - CZP 400 mg Q4W, SC - vs. PBO	- Clinical response at induction (W6): 64% - Clinical remission at W26: CZP 48% vs. PBO 29% (*p <* 0.001).
	CZP	WELCOME (159)	III	539	Active CD, TNFi IR (maintenance)	Open-labeled induction (*n =* 539): CZP 400 mg at W0, 2, 4, SC - Patient with clinical response were randomized at W6 for open-labeled maintenance (*n =* 329): - CZP 400 mg Q2W, SC - vs. CZP 400 mg Q4W, SC - vs. PBO	- Clinical response at induction (W6): 62% - Clinical response at W26: CZP Q2W 37% vs. CZP Q4W 40% (*p =* 0.55). - Clinical remission at W26: CZP Q2W 30% vs. CZP Q4W 29% (*p =* 0.81).
IL-17i	BRO	(160)	II	130	Active CD (induction)	BRO 210 mg vs. 350 mg vs. 700 mg Q4W for 4W, SC vs. PBO	- Early termination due to worsening CD in active treatment groups, *n =* 130 analyzed at termination- Clinical response at W6: BRO 210 mg 16% vs. 350 mg 27% vs. 700 mg 15% vs. PBO 13%. - Clinical remission at W6: BRO 210 mg 3% vs. 350 mg 15% vs. 700 mg 9% vs. PBO 13%.
	UST	CERTIFI (161)	IIb		Active CD, TNFi IR (induction and maintenance)	Induction W0-8 (*n =* 539): - UST 1, 3, 6 mg/kg, SC - vs. PBO - Maintenance 8–36W (re-randomized at W8): - UST 90 mg at W8 and 16, SC - vs. PBO	- Clinical remission at W6 (induction): UST6 mg/kg 39.7% vs. PBO 23.5% (*p =* 0.005) NS for other UST doses Maintenance for those responded to induction, *n =* 145 - Clinical response at W22: UST 69.4% vs. PBO 42.5% (*p <* 0.05) - Clinical remission at W22: UST 42% vs. PBO 27% (*p <* 0.05)
IL-12/23i	UST	IM-UNITI (162)	III	397	UNITI-1: active CD, TNFi IR (*n =* 741). UNITI-2: active CD, immunomodulator IR (*n =* 628). IM-UNITI: Who had clinical response in UNITI-1 and 2 (*n =* 397)	Induction W0-8 (UNITI-1 or 2): - UST 130 mg, SC - vs. UST 6 mg/kg, SC - vs. PBO - Maintenance W8-44: - UST 90 mg/8W, SC - vs. UST 90 mg/12W, SC - vs. PBO	Induction: Clinical remission at W8:- UNITI-1 UST 6 mg/kg 38% (*p <* 0.001) vs. UST 130 mg 34% (*p =* 0.001) vs. PBO 20% (all comparison vs. PBO)- UNITI-2 UST 6 mg/kg 58% (*p <* 0.001) vs. UST 130 mg: 47% (*p =* 0.001) vs. PBO 32% (all comparison vs. PBO) Maintenance (IM-UNITI) - Clinical response: UST 90 mg Q8W 59% (*p =* 0.02) vs. UST 90 mg Q12W 58% (*p =* 0.03) vs. PBO 44% (all comparison vs. PBO) - Clinical remission UST 90 mg Q8W 53% (*p =* 0.02) vs. UST 90 mg Q12W 49% (*p =* 0.03) vs. PBO 36% (all comparison vs. PBO)
IL-23i	RZB	(163)	II	121	Active CD, TNFi IR (induction)	- RZB 600 mg Q4W - vs. RZB 200 mg Q4W - vs. PBO	- Clinical response at W12: RZD 600 mg 42% (*p =* 0.0366) vs. RZD 200 mg 37% (*p =* NS) vs. PBO 21% (all comparison vs. PBO) - Clinical remission at W12: RZD 600 mg 37% (*p =* 0.025) vs. RZD 200 mg: 24% (*p =* NS) vs. PBO 15% (*p =* 0.049) (all comparison vs. PBO)
JAKi	TOF	(164)	II	139	Active CD (induction)	TOF 15, 5, 5 mg BD, PO vs. PBO	- Clinical response W4: TOF 15 mg 46% (*p =* 0.467) vs. 5 mg 58% (*p =* 0.466) vs. 5 mg: 36% (p≥0.999) vs. PBO 47% (all comparison vs. PBO).- Clinical remission at W4: TOF 15 mg 14% (*p =* 0.540) vs. 5 mg 24% (*p =* 0.776) vs. 5 mg 31% (*p =* 0.417) vs. PBO 21% (all comparison vs. PBO).
	TOF	(165)	IIb	280 (induction) 180 (maintenance)	Active CD, % prior TNFi (induction and maintenance)	Induction 0-8W (*n =* 280) - TOF 10 mg BD, PO - vs. TOF 5 mg BD, PO - vs. PBO - Maintenance 8–26W for those responded to TOF induction (*n =* 180): - TOF 10 mg BD, PO - vs. TOF 5 mg BD, PO - vs. PBO	- Clinical remission at W8 (induction): TOF 10 mg 43% (*p =* 0.392) vs. TOF 5 mg 44% (*p =* 0.325) vs. PBO 36.7% (all comparison vs. PBO). - Clinical remission at W26 (maintenance): TOF 10 mg 56% (*p =* 0.13) vs. TOF 5 mg 40% (*p =* NS) vs. PBO 38.1%. (all comparison vs. PBO).
	FIL	FITZROY (166)	II	174	Active CD, 27% prior bowel resection, 58% prior TNFi (induction)	FIL 200 mg/day, PO vs. PBO	- Clinical remission at W10: - FIL 47% vs. PBO 36.7%. (*p =* 0.0077)
	UPA	CELEST (167)	II	220	Active CD (induction and maintenance)	Induction W0-16: UPD 3, 6, 12, 24 mg BD or 24 mg/day vs. PBO Maintenance W16-52: UPD 3, 6, 12, 24 mg BD or 24 mg/day No PBO control	- Clinical remission at W16: UPA 3 mg 13% (NS) vs. 6 mg 27% (*p <* 0.1 vs. PBO) vs. 12 mg 11% (NS) vs. 24 mg: 22% (NS) vs. 24 mg/day 14% (NS) vs. PBO 11%. (all comparison vs. PBO) - Endoscopic remission at W16: - UPA 3 mg 10% (*p <* 0.1) vs. 6 mg 8% (NS) vs. 12 mg 8% (*p <* 0.1) vs. 24 mg 22% (*p <* 0.01) vs. 24 mg/day 14% (*p <* 0.05) vs. PBO 0%. (all comparison vs. PBO). - Maintenance: Efficacy was maintained for most endpoints through week 52

*ADA, adalimumab; AZA, azathioprine; BD, Twice daily; BRO, brodalumab; BW, Body weight; CD, Crohn's Disease; CZP, certolizumab; FIL, filgotinib; Gp, Group; IFX, infliximab; IL, Interleukin; i, inhibitor; IR, inadequate responder; IV, intravenous; JAKi, Janus kinase inhibitor; NS, not statistically significant; PBO, placebo; Q, every; RCT, Randomized control trial; RZB, risankizumab; TNF, tumor necrosis factor; inhibitor; TOF, tofacitinib; UPA, upadacitinib; UST, ustekinumab; vs., versus; W, week; yr, year*.

Vedolizumab (VZD) is a monoclonal antibody targeting α4β7 integrin, which reduces lymphocytes trafficking to the gastrointestinal tract by blocking lymphocyte surface α4β7 binding to the mucosal addressin cell adhesion molecule-1 (MAdCAM-1). The efficacies of VZD in induction and maintenance in CD have been demonstrated in the GEMINI-2 (172) and GEMINI-3 trials (173) (Table 1). In a meta-analysis involving 1716 patients with CD, VZD was more effective than placebo for inducing clinical remission (RR 1.71 [95% confidence interval, CI: 1.25, 2.34], *p* = 0.0008), and maintaining clinical remission (RR 1.75 (95% CI: 1.25, 2.44), *p* <0.001).

Ustekinumab (UST) is an antibody targeting the IL-12/23 p40 subunit. The efficacy of UST in inducing remission in CD has been shown in UNITI-1 and UNITI-2 trials in patients with inadequate response to TNFi, and without prior TNFi failure, respectively. Responders from both studies were randomized to the IM-UNITI maintenance study and demonstrated significantly higher clinical remission rates [high dose: 53%, *P* = 0.005; low dose: 49%, *P* = 0.04)] compared to placebo (36%) at week 44 (162). There is no head-to-head study comparing efficacies between TNFi, VZD and UST. The choice of biological treatment is a shared decision between clinician and patient, and according to the individual risk–benefit preferences.

Risankizumab (RZB), an IL-23/p19 inhibitor met the primary remission induction endpoints in CD in two phase III RCTs, ADVANCE (NCT03105128) and MOTIVATE (NCT03104413) (174). Patients in remission from ADVANCE and MOTIVATE were recruited to the Phase III open-label maintenance study, FORTIFY, showing RZB 360 mg every 8 weeks achieved the co-primary endpoints of clinical remission and endoscopic response at 52 weeks (175).

Blocking IL-17, however, has not been effective in CD. In a phase II trial evaluating safety and efficacy of brodalumab (BRO), a monoclonal antibody targeting IL-17 receptor, the primary induction endpoint was not met. The trial was terminated early due to a disproportionate number of cases of worsening CD (160). In a phase II RCT, two doses of 10 mg/kg secukinumab (SEC) given intravenously on days 0 and 22, failed to meet the primary endpoint and had more adverse events compared to placebo (176). However, the use IL-17i is not associated with increased incidence of IBD. Data from the SEC development program pooling 7,355 patients with a cumulative exposure of 16,227 person-years of patients exposed to SEC for PsO, PsA or SpA, no increase in exposure adjusted incidence rates of IBD was observed (15). Similarly, events of IBD remained low in the ixekizumab development program that pooled data from 15 RCTs in PsO and PsA (177).

Phase II RCT results for the Janus kinase inhibitor (JAKi), upadacitinib (UPA), in CD are promising. Endoscopic but not clinical remission increased with dose during the induction period (167). However, in a phase II trial for JAKi, tofacitinib (TOF), no statistically significant differences in clinical responses between TOF and placebo were observed at week 4 (164) (Table 2).

### Medical Treatment for UC

Oral 5-ASA (5-aminosalicylic acid) is the standard therapy for induction in mild-to-moderately active UC. For those failing 5-ASA or with moderate-to-severe UC, a short 6- to 8-week course of oral corticosteroid is indicated. 5-ASA and thiopurines can be used as maintenance therapy. Like the treatment strategy for CD, early escalation to biologic therapies should be considered for those who failed induction therapy with corticosteroid, or failed maintenance with immunomodulators, and those with poor prognostic factors. TNFi [IFX, ADA, golimumab (GOL)], α4β7 integrin inhibitor (VZD) and IL12/23i (UST) are approved treatments for induction and maintenance of UC (Table 3). A combination of TNFi with an immunomodulator is more effective. In the UC-SUCCESS trial, patients treated with IFX and AZA were more likely to achieve corticosteroid-free remission at 16 weeks than those receiving either monotherapy (181). In a head-to-head study (VARSITY) comparing TNFi and VZD in patients with moderate-to-severe UC, 769 patients with moderate-to-severe active UC were randomized to receive VDZ or ADA (185). Only 26% of patients in either group were on concomitant immunomodulators. At week 52, a higher percentage of patients achieved clinical remission (31.3 vs. 22.5%; *P* = 0.006), and endoscopic improvement (39.7 vs. 27.7%; *P* <0.001) in VDZ compared to ADA group. Whilst corticosteroid-free clinical remission occurred at a higher rate in the group receiving ADA compared to VDZ (21.8 vs. 12.6%; difference, −9.3 percentage points; 95% CI, −18.9 to 0.4). Despite a slight superiority of VDZ over ADA, more data are pending for consistency and class effect. The choice of biologics is, again, a shared decision between between clinician and patient.

**Table 3:** Evidence from clinical trials for class of therapeutic options for ulcerative colitis.

**Class of drug**	**Agent**	**Trial acronyms**	**RCT Phase**	**Sample size**	**Patient population**	**Treatment vs. comparison**	**Outcomes**
TNFi (mAb)	ADA	Ultra 1 (178)	III	186	Active UC, despite corticosteroid and/or immunomodulators (induction)	- ADA 160 mg at W0, 80 mg at W2, 40 mg at W4 and 6, SC - Vs. ADA 80 mg at W0 and 2, 40 mg at W4 and 6, SC - Vs. PBO	- Clinical remission at W8: ADA160/80 18.5% (*p =* 0.031 vs. PBO) vs. ADA 80/40 10.0% (*p =* 0.833 vs. PBO) vs. 9.2% PBO
	ADA	Ultra 2 (179)	III	494	Active UC, despite corticosteroid and/or immunomodulators 40% prior TNFi (induction and maintenance)	- ADA 160 mg at W0, 80 mg at W2, and then 40 mg Q2W, SC - Vs. PBO	- Clinical remission at W8: - ADA 16.5% vs PBO 9.3% (*p =* 0.019) - Clinical remission at W52 ADA 17.3% vs PBO 8.5% (*p =* 0.004) - Better response in TNFi naïve patients
	IFX	ACT I (180)	III	364	Active UC despite corticosteroid and/or thiopurines (induction and maintenance)	- IFX 5 mg or 10 mg/kg at W0, 2, 6, 14, 22, 30, 38, and 46, IV - Vs. PBO	- At W8, higher clinical response in IFX groups: IFX 10 mg/kg vs. IFX 5 mg/kg vs. PBO: 61.5% vs. 69.4% vs. 37.2%, (all *p <* 0.001 compared to PBO). - At W8, higher clinical remission in IFX groups: IFX 10 mg/kg vs. IFX 5 mg/kg vs. PBO: 32% vs. 38.8% vs. 14.9%, (all *p <* 0.001 compared to PBO). - Remission rate at W54: IFX 10 mg/kg vs. IFX 5 mg/kg vs. PBO (34.4% vs. 34.7% vs. 16.5%), (all *p =* 0.001 compared to PBO).
	IFX	ACT II (180)	III	364	Active UC despite corticosteroid and/or thiopurines and 5-ASA (induction and maintenance)	- IFX 5 mg or 10 mg/kg at W0, 2, 6, 14, and 22, IV - Vs. PBO	- At W8, higher clinical response in IFX groups: IFX 10 mg/kg vs. IFX 5 mg/kg vs. PBO: 69.2% vs. 64.5% vs. 29.3%, (all *p <* 0.001 compared to PBO). - At W8, higher clinical remission in IFX groups: IFX 10 mg/kg vs. IFX 5 mg/kg vs. PBO: 27.5% vs. 33.9% vs. 5.7%, (all *p <* 0.002 compared to PBO). - Remission rate at W30: IFX 10 mg/kg vs. IFX 5 mg/kg vs. PBO: 35.8% vs. 25.6% vs. 10.6%, all *p =* 0.001 compared to PBO.
	IFX	US-SUCCESS (181)	III	239 (planned 600)	Active UC (induction)	- IFX 5 mg/kg at weeks 0, 2, 6, and 14, IV + AZA 2.5 mg/kg/day, PO - Vs. IFX alone - Vs. AZA alone	- Study terminated early before enrolment target (intermittent IFX regimen raised concern for injection site reaction in another study) - Corticosteroid-free remission at W16: IFX+AZA 39.7% vs. IFX alone 22.1% (*p =* 0.017) vs. AZA alone 23.7% (*p =* 0.032). - Mucosal healing at W16: IFX+AZA 62.8% vs. IFX alone 54.6% (p = 0.295) vs. AZA alone 36.8% (*p =* 0.001).
	GOL	PURSUIT - SC (182)	III	761	Active UC despite corticosteroid and/or immunomodulators (induction)	- GOL 400 mg at W0, then 200 mg at W2, SC - Vs. GOL 200 mg at W0, then 100 mg at W2, SC - Vs PBO	- Clinical response at W6: GOL 400/200 54.9% vs. GOL 200/100 51% vs. PBO 30.3% (all *p <* 0.001 vs. PBO) - Clinical remission at W6: GOL 400/200 17.9% vs. GOL 200/100 17.8% vs. PBO 6.4% (all *p <* 0.001 vs. PBO) - Mucosal healing at W6: GOL 400/200 45.1% vs. GOL 200/100 42.3% (*p =* 0.0014 vs. PCB) vs. PBO 28.7% (all *p <* 0.001 vs. PBO)
	GOL	PURSUIT - MAINTENANCE (183)	III	464	UC patients responded to GOL induction (maintenance)	- GOL 100 mg Q4W, SC - Vs. GOL 50 mg Q4W, SC - Vs. PBO	- Clinical response maintained at 54W: GOL100 49.7% (*p =* 0.01) vs. GOL50 47% (*p <* 0.001) vs. 31.2% PBO (all comparison vs. PBO) - Clinical remission at both W30 and W54: GOL100 27.8% (*p =* 0.004) vs. GOL50 23.2% (NS) vs. 15.6% PBO (all comparison vs. PBO) - Mucosal healing at both W30 and W54: GOL100 42.4% (*p =* 0.002) vs. GOL50 41.7% (*p =* 0.011) vs. 26.6% PBO (all comparison vs. PBO)
α4β7 integrin inhibitor	VDL	GERMIN I (184)	III	Induction = 886 Maintenance = 373	- Induction: active UC despite corticosteroid and/or immunomodulators (48.2% prior TNFi) maintenance: patients responded to induction phase	- VDL 300 mg Q4W, IV - Vs. VDL 300 mg Q8W, IV - Vs. PBO - (both induction and maintenance)	- Induction phase at W6: °Clinical response: VDL 47% vs. PBO 25.5%, *p <* 0.001 °Clinical remission: VDL 16.9% vs. PBO 5.4%, *p =* 0.001 °Mucosal healing: VDL 40.9% vs. 24.8%, *p =* 0.001 - Maintenance phase at W52: °Clinical remission: VDLQ4 44.8% vs. VDLQ8 41.8% vs. PBO 15.9% (all *p <* 0.001 vs. PBO)
							°Mucosal healing: VDLQ4 56% vs. VDLQ8 51.6% vs. PBO 19.8% (all *p <* 0.001 vs. PBO)
	VDL vs. ADA	VARSITY (185)	III	769	- Active UC despite corticosteroid, or immunomodulators (Non TNFi failure) - 21% Prior TNFi exposure - 26% concomitant immunomodulators	- VDL 300 mg W0, 2, 6, 14, 22, 30, 38, and 46, IV - ADA 40 mg 160 mg at W0, 80 mg at W2, then 40 mg Q2W till W50, SC	- Clinical response at W52: VDL 31.3% vs. ADA 22.5%, *p =* 0.006 - Endoscopic improvement at W52: VDL39.7% vs. ADA 27.7%; *P <* 0.001. - Corticosteroid-free remission at W52: VDL 12.6% vs. ADA 21.8%, NS
JAKi	TOF	OCTAVE Induction-1 (186)	III	598	Active UC despite immunomodulators/ TNFi 74% TNFi failure	- TOF 10 mg BD, PO - Vs. PBO	- Clinical remission at W8: TOF 18.5% vs. PBO 8.2%, *p =* 0.007 - Mucosal healing at W8: TOF 31.3% vs. 15.6%, *p <* 0.001
		OCTAVE Induction-2 (186)	III	541	- Active UC despite immunomodulators/ TNFi - 70% TNFi failure	TOF 10 mg BD, PO - Vs. PBO	- Clinical remission at W8: TOF 16.6% vs. PBO 3.6%, *p <* 0.001- Mucosal healing at W8: TOF 28.4% vs. 11.6%, *p <* 0.001
		OCTAVE - Sustain (186)	III	593	- Patients who has a clinical response in OCTAVE 1 and 2	- TOF 10 mg BD, PO - Vs. TOF 5 mg BD, PO - Vs. PBO	- Clinical remission at W52: TOF10 40.6% vs. TOF5 34.3% vs. PBO 11.1%, (all *p <* 0.001 vs. PBO) - Mucosal healing at W52: TOF10 45.7% vs. TOF5 37.4% vs. PBO 13.1%, (all *p <* 0.001 vs. PBO)
	UPA	AbbVie UPA UC development program	III	>1300	NCT02819635, NCT03653026, NCT03006068	No details yet	- Preliminary: met primary endpoints of clinical response, remission, endoscopic improvement, and response - No detail yet

*ADA, adalimumab; AS, ankylosing spondylitis; axSpA, axial spondyloarthritis; AZA, azathioprine; BD, twice daily; CI, confidence interval; CZP, certolizumab; GOL, golimumab; Gp, group; HR, hazard ratio; IFX, infliximab; IR, incidence rate ratio; IV, intravenous; JAKi, Janus kinase inhibitor; M, month; PO, per oral; Q, every; NS, not statistically significant; PBO, placebo; SC, subcutaneous; SEC, secukinumab; TNFi, tumor necrosis factor inhibitor; TOF, tofacitinib; UPA, upadacitinib; VDL, vedolizumab; Vs., versus; W, week; Yr, year*.

Due to the ineffectiveness of IL-17i for CD, there have not been trials for their use in UC. As for IL-23i, there is an ongoing phase II/III trial of RZB for UC (NCT03398148).

In contrast to CD, the JAKi, TOF, was approved for use in moderate-to-severe UC based on three pivotal phase III OCTAVE studies, showing a significantly greater percentage of clinical remission at week 8 for induction, and remission at week 52 for maintenance in TOF compared to placebo group (186). UPA met the clinical remission, endoscopic improvement and histological improvement endpoints in a phase III induction trial for moderate-to-severe UC (187).

### Medical Treatment for Uveitis

Prompt control of inflammation using topical corticosteroid is the first-line treatment for anterior uveitis in SpA. Typically, prednisolone acetate 1% eye drops are used as for severe AAU while milder corticosteroids such as dexamethasone 0.1% may be used for maintenance. A mydriatic drug is often prescribed together to reduce the development of posterior synechia and reduce pain from ciliary body spasm. Periocular corticosteroid injections or intravitreal implants can be used for more chronic cases. Adverse effects of corticosteroid in the eyes include cataract and ocular hypertension in up to 30% of patients. Oral corticosteroids may be used for acute management of severe and sight-threatening posterior uveitis such as vasculitis and cystoid macular edema, however, immunotherapy should be considered early in these cases to reduce recurrences (188). Traditional immunomodulators such as sulfasalazine (SSZ) and MTX may be tried although few data have supported their efficacy. Monoclonal antibody-TNFi including IFX, ADA, GOL and CZP are considered as effective treatment options for both acute flares and reducing recurrences of AAU (189). We summarize the major RCTs of therapeutic options for uveitis in Table 4. In a *post-hoc* analysis pooling data from four RCTs with TNFi in AS, the frequency of AAU flares was substantially lower among IFX or etanercept (ETN) treated than placebo treated patients. Lower frequency of AAU flares was seen in the open-labeled extension phase compared to the placebo phase of the trial (TNFi: 6.8 flares per 100 patient-years compared to PBO: 15.6 per 100 patient-years, *p* = 0.01) (198). ADA is the only TNFi licensed for treatment of non-infectious uveitis in adult following favorable results in 2 phase III RCTs. In the VISUAL I study, patients with active non-infectious intermediate, posterior uveitis or panuveitis were randomized to receive ADA or placebo after a prednisolone burst (60 mg) with tapering course. Patients treated with ADA were less likely than those treated with placebo to have treatment failure (hazard ratio, HR, 0.50; 95% CI, 0.36 to 0.70; *P* <0.001). The VISUAL II study recruited 226 patients with inactive, non-infectious intermediate, posterior, or panuveitis controlled by 10–35 mg/day of prednisone were randomized to ADA vs. placebo. All patients underwent a mandatory prednisolone tapering to 0 mg by week 19. The time to treatment failure was significantly longer in ADA compared to placebo arm (median >18 months vs. 8.3 months; HR 0.57, 95% CI 0.39–0.84; *p* = 0.004) (191). ADA is also licensed for juvenile idiopathic arthritis-related uveitis. In an open-label study in 93 AS patients with history of uveitis, GOL reduced uveitis flares compared to patients' historical control 12-month prior to initiation of GOL (192). There is an ongoing phase III 96-week open-label study for CZP in 115 patients with axial SpA and recurrent uveitis. In the 48-week interim analysis of 85 patients, uveitis flares were substantially reduced during the CZP treated period compared to the historical rates (64.0 and 31.5% respectively) (193). The use of ETN in the management of uveitis has diminished in favor of other TNFi because of its weaker ability in preventing flares.

**Table 4:** Evidence from clinical trials for class of therapeutic options for uveitis.

**Class of drug**	**Agent**	**Trial acronyms**	**Trial Phase**	**Sample size**	**Patient population**	**Treatment vs. comparison**	**Outcomes**
TNFi	ADA	VISUAL-1 (190)	RCT, III	217	Active non-infectious intermediate uveitis, posterior uveitis, or panuveitis despite corticosteroid	- ADA loading 80 mg, then 40 mg Q2W, SC - Vs. PBO	- FU: till 80w or pre-specified events of treatment failure is reached. - Longer median time to treatment failure, ADA vs PBO (24w vs. 13w) - ADA less likely than PBO group to have treatment failure (HR 0.50; 95% CI: 0.36-0.70; *P <* 0.001).
	ADA	VISUAL-2 (191)	RCT, III	229	Inactive, non-infectious intermediate, posterior, or panuveitis requiring prednisolone for maintenance	- ADA loading 80 mg, then 40 mg Q2W, SC - Vs. PBO	- FU: till 80w or at treatment failure event - Long time to treatment failure, ADA vs PBO (10.2m vs. 4.8m) - ADA less likely than PBO group to have treatment failure (HR 0.57; 95% CI: 0.39-.84; *P =* 0.004).
	GOL	GO-EASY (192)	Open label, non-randomized	93	- AS patients (55% TNFi-naive, 27% history of uveitis)	All: GOL 50 mg monthly - VS. historical control (flare rates in previous yr)	- Lower risk of uveitis flare in GOL vs. historical rates (2.2 vs. 11.1 per 100 patient-years, rate-ratio 0.20, 95% CI 0.04–0.91).
	CZP	(Abstract only) (193)	Open label, non-randomized, IV	115 enrolled (85 in interim analysis)	Active axSpA, HLAB27 positive, having history of recurrent uveitis	- All: 400 mg at W0, 2, 4, then 200 mg Q2W till W96 - Vs. historical control	- Interim analysis of 85 patients completed W48 - Few flares CZP vs. historical rates (Poisson-adjusted IR: 0.2 vs 1.5, *p <* 0.001).
IL-17i	SEC	3 RCTs: SHIELD, INSURE, ENDURE (194)	RCT, III	274	Behçet's uveitis = 118 (SHIELD) Active non-infectious active uveitis = 31 (INSURE) Inactive non-infectious uveitis = 125 (ENDURE)	- Varies dosing: - SEC loading (150 mg or 300 mg), then Q2W-Q4W - Vs. PBO	- SHIELD: completed, primary endpoint not met - INSURE: terminated early - ENDURE: completed, planned analysis dropped - No statistically significant differences in uveitis flares, SEC vs. PBO in all 3 RCTs
	SEC	(195)	II	37	Active non-infectious intermediate uveitis, posterior uveitis, or panuveitis, requiring corticosteroid sparing therapy	- SEC 30 mg/kg Q4W, IV for 2 doses, (Group 1) - Vs. SEC 10 mg/kg Q2W, IV for 4 doses, (Gp 2) - Vs. SEC 300 mg Q2W, SC for 4 doses, (Gp 3)	- Higher response rate in higher dose compared to lower dose regimen on day 57. - Responder rates (Gp 1: 72.7% vs. Gp2: 61.5% vs. Gp3: 33.3%, statistically significant Gp 1 vs. Gp3) - Remission rates (Gp1: 27.3% and Gp2: 38.5% vs. Gp3: 16.7%, NS)
IL-12/23i	UST	STAR (196)	II	8 enrolled	Active sight-threatening active intermediate uveitis, posterior uveitis, or panuveitis	- 90 mg, SC at W0,4 and 8 vs 260-520 mg (weight-based dose), IV at W0 then 90 mg, SC at W8	- Completed, awaiting analysis and publication of results
	UST	STELABEC-2 (197)	II	16	Active posterior uveitis and/or panuveitis and/or retinal vasculitis in patients with Behçet's disease	- 90 mg, SC at W0, W4, and W16. Patients with response will receive 90 mg, SC at W28 and W40	- Ongoing

*ADA, adalimumab; AS, ankylosing spondylitis; axSpA, axial spondyloarthritis; CI, confidence interval; CZP, certolizumab; GOL, golimumab; Gp, group; HR, hazard ratio; IR, incidence rate ratio; IV, intravenous; M, month; Q, every; NS, not statistically significant; PBO, placebo; SC, subcutaneous; SEC, secukinumab; TNFi, tumor necrosis factor inhibitor; UST, ustekinumab; Vs., versus; W, week; Yr, year*.

Despite implicated in the pathogenesis of uveitis, inhibiting IL-17A was not effective for uveitis. In three RCTs, SEC failed to meet the primary efficacy endpoints (194). In another RCT comparing three doses of SEC, statistical higher response rates and remission on day 57 for the high dose regimen (30 mg/kg intravenously Q2W for 4 doses) was seen compared to the other two lower dose regimens, suggesting a higher dose intravenous regimen may be required to deliver SEC in therapeutic concentrations (195). Results are awaiting for two trials using UST in active sight-threatening uveitis (STAR) (196) and Behçet uveitis (STELABEC) (197), which may provide insight for its potential use in PsA related uveitis.

Minimal data exist for use of JAKi in uveitis. One phase 2 RCT evaluating filgotinib (FIL) in patients with active non-infectious uveitis (NCT03207815) is ongoing.

## Management of PSA With Consideration of Extra-Articular Manifestations

Given the heterogeneity in manifestations, enhanced collaboration between disciplines are required to deliver optimal care for PsD (199). While collaborations between rheumatologists and dermatologists are increasing (200), collaborations with gastroenterologists and ophthalmologists have traditionally been weaker. Apart from setting up combined clinics, collaborations between disciplines can take other forms as determined by needs and circumstances of different institutions. Minimally, identifying key stakeholders specializing in the care of PsA patients and keeping them in close communication over the management plan is essential. These collaborations serve both clinical and educational needs. Close collaboration between the various disciplines will help in early diagnosis of the various manifestations, providing expert advice on choice of therapeutics to create a patient-centric, individualized care plan for the heterogeneous manifestations. Often, the therapeutics will need to cover multiple domains, but the predominant domain should drive the therapeutic option of choice in the shared decision-making process.

For severe IBD in the setting of PsA with peripheral manifestations, traditional immunomodulators can be considered for maintenance. TNFi (monoclonal antibodies) is a better option for patients with axial arthropathy. UST is effective for IBD but is less effective on peripheral arthritis as compared to TNFi or IL-17i, and ineffective for axial arthropathy. While IL-17i is an effective treatment for both peripheral and axial arthropathy, and probably does not increase the risk of IBD, it is not recommended for patients with underlying active IBD, due to its possibility of exacerbating pre-existing disease. IL-23i may be promising for IBD but its use requires caution in patients with predominant axial arthropathy. JAKi is effective for UC, peripheral and axial arthropathy, but may exacerbate CD. VDZ is effective for both CD and UC but has no indication for all other manifestations in PsA. With these considerations, TNFi (monoclonal antibodies) with or without concomitant immunosuppressants would be the best option for PsA patients with IBD. IBD is a chronic relapsing condition, and often requires higher doses of TNFi for induction than arthritis alone. Collaboration between rheumatologist and gastroenterologist is invaluable to ensure the optimal choice of treatment regimen.

Uveitis can be serious and sight threatening. Patients with symptoms of possible uveitis should have access to ophthalmology care promptly and given appropriate treatment for uveitis. Uveitis can arise even when arthritis is under control; it may manifest either suddenly or insidiously. It is important that patients are educated to be aware of the symptoms of uveitis and seek appropriate care when the needs arise. Care models like enquiry hotline, early referral or walk-in ophthalmology clinics are examples that may facilitate early diagnosis. For subsequent management, collaboration between rheumatologist and ophthalmologist is essential to ensure regular assessment of response to therapy and to modify management accordingly. If uveitis fails to respond to topical corticosteroids, or fails to be weaned, or is severe at the onset, an escalation to either conventional immunomodulators or biological agents should be considered. The use of systemic corticosteroid is best avoided, given the risk of severe PsO flare upon its withdrawal. For patients with peripheral musculoskeletal manifestations (peripheral arthritis, enthesitis and dactylitis), MTX, SSZ or leflunomide (LEF) can be tried for maintenance, but an early escalation to TNFi (monoclonal antibodies) should be considered if these options fail. Traditional immunomodulators are not effective for axial arthropathy, thus for patients with active axial arthropathy TNFi (monoclonal antibodies) would be a good choice. Some patients may require higher or more frequent doses of TNFi especially for severe uveitis, highlighting again the importance of collaboration between rheumatologist and ophthalmologist for drug titration. IL-17i is an effective treatment for axial arthropathy, but SEC may not be effective for AAU at standard dose, and more data is still needed to inform the use of other IL-17i.

All in all, detailed considerations of all domains and extra-articular manifestations are necessary to formulate the best therapeutic option. Multi-disciplinary collaborative care models are advocated for optimal care for patients with PsA, and especially so for those who present with co-morbidities.

## Author Contributions

AlC and YL conceptualized the project. AlC, TC, and YL collected relevant data for review and drafted the manuscript. AnC, WC, GA, and YL critically appraised the content of manuscript. All authors approved the final version of manuscript.

## Funding

YL was supported by the National Medical Research Council, Singapore (NMRC/CSA-Inv/0022/2017). The funding sources had no role in the views expressed in this review.

## Conflict of Interest

The authors declare that the research was conducted in the absence of any commercial or financial relationships that could be construed as a potential conflict of interest.

## Publisher's Note

All claims expressed in this article are solely those of the authors and do not necessarily represent those of their affiliated organizations, or those of the publisher, the editors and the reviewers. Any product that may be evaluated in this article, or claim that may be made by its manufacturer, is not guaranteed or endorsed by the publisher.
